# Effects of visual deprivation on the injury of lower extremities among functional ankle instability patients during drop landing: A kinetics perspective

**DOI:** 10.3389/fphys.2022.1074554

**Published:** 2022-12-12

**Authors:** Lingyue Meng, Lintao Kong, Lingyu Kong, Qiuxia Zhang, Jianzhong Shen, Yuefeng Hao

**Affiliations:** ^1^ Physical Education and Sports School, Soochow University, Suzhou, China; ^2^ Experimental Primary School, High Tech Zone Science and Technology City, Suzhou, China; ^3^ Rehabilitation Center, Shanghai Yongci Rehabilitation Hospital, Shanghai, China; ^4^ Orthopedics and Sports Medicine Center, The Affiliated Suzhou Hospital of Nanjing Medical University, Suzhou, China

**Keywords:** injury risk, motor strategies, lower extremities, biofeedback, drop landing

## Abstract

**Background:** The ankle is prone to injury during drop landing with usual residual symptoms, and functional ankle instability (FAI) is the most common. Vision guarantees the postural stability of patients with FAI, and visual deprivation (VD) increases their risk of injury when completing various movements. This study explored injury risk during drop landing in patients with FAI under VD through the kinetics of lower extremities.

**Methods:** A total of 12 males with FAI participated in the study (age, 23.0 ± 0.8 years; height, 1.68 ± 0.06 m; weight, and 62.2 ± 10.4 kg) completed single-leg drop landings under visual presence (VP) and VD conditions. Ground reaction force (GRF), time to peak GRF, joint torque, and vertical length variation (ΔL) were measured.

**Results:** Significant effects were detected in the group for time to peak lateral GRF (*p* = 0.004), hip extensor torque (*p* = 0.022), ankle plantarflexion torque (*p* < 0.001), ankle varus torque (*p* = 0.021), lower extremity stiffness (*p* = 0.035), and ankle stiffness (*p* < 0.001). Significant effects of conditions were detected for vertical GRF, time to peak vertical and lateral GRF, loading rate, hip extensor torque, knee extensor torque, hip varus torque, knee varus torque, lower extremity stiffness, and ankle stiffness (*p* < 0.05). ΔL was affected by VD with a significant difference (*p* < 0.001).

**Conclusion:** In patients with FAI, an unstable extremity has a higher injury risk than a stable extremity, and VD increases such risk. However, because the influence of the central nervous system on hip strategy is also affected, the effect on the unstable extremity is more significant and more likely to result in injury. Deepening the squat range may be an effective preventive measure for reducing injury risk of unstable extremities during drop landing.

## Introduction

Ankle sprain is the most common and severe injury in sports (Mckay et al., 2001). Repeated ankle injuries lead to chronic ankle instability (CAI), which is mainly caused by old injuries to ankle ligaments. CAI symptoms include local pain, decreased postural control, and decreased proprioception. CAI can be divided into functional ankle instability (FAI) and mechanical ankle instability (MAI) (Yu et al., 2022). Compared with individuals without a history of ankle injury, individuals with ankle injury history reported significant deficits in foot proprioception and in their static and dynamic balance (Alghadir et al., 2020), and over half of them did not seek professional treatment (Mckay et al., 2001). Muscle force and movement control are impaired if the injured ankle does not receive prompt and professional rehabilitation treatment, resulting in FAI (Freeman, 1965; Kim et al., 2021). Patients with FAI are almost five times more likely to sustain repeated episodes of ankle instability and recurrent ankle sprains than are healthy individuals (Mckay et al., 2001; Kaminski et al., 2003; Raymond et al., 2012; Gouttebarge et al., 2017), leading to irreversible damage to ankle stability (Hong et al., 2021).

Human sensory systems play essential roles in adjusting postural control and preventing injury, including the proprioception (Horak et al., 1990; Delahunt et al., 2006; Imbimbo et al., 2021; Oosterman et al., 2021), vestibular (Gosselin and Fagan, 2014; Brown et al., 2021; Kalinina et al., 2021; Morawietz and Muehlbauer, 2021), and visual systems. Patients with FAI usually exhibit proprioceptive impairment, which is the principal consideration for recurrent sprains, and visual information provides positional sensation and feedback to the motor system to offset deficits in proprioception and maintain their postural stability (Doherty et al., 2014; Toth et al., 2017; Raffalt et al., 2019; Han et al., 2022). Therefore, once patients with FAI transit from a visual presence (VP) to a visual deprivation (VD) condition, visual information cannot be transmitted and their postural stability becomes more challenging (Raffalt et al., 2019; Rosen et al., 2021). Some studies related to gait in VD suggest that VD is the main cause of falls in elderly individuals and of increased injury rates in athletes (Isakov and Mizrahi, 1997; Duggan et al., 2017; McDonough et al., 2017). Nonetheless, the research on VD among patients with FAI is relatively deficient, especially when patients are completing challenging tasks.

In addition, drop landing is one of the most challenging motion forms in daily physical activities and professional training. It is commonly seen in sports such as basketball and volleyball, and even in the process of stepping down during daily life. Ankle injuries from drop landing can be as high as 45%. Some studies have shown that patients with FAI have significantly decreased local muscle activity and deficits in postural instability (Delahunt et al., 2006; Rosen et al., 2013). Postural stability during drop landing requires rapid visual and position sensation integration. However, protective strategies in patients with FAI during drop landing with VD remain unclear, and the risk factors associated with VD in patients with FAI must be identified. This paper aimed to characterize the effects of VD and compensatory mechanisms under VD in patients with FAI during drop landing, through the kinetics of lower extremities. It hypothesized the following: 1) differences exist in the kinetics of patients with FAI during drop landing on stable and unstable extremities, 2) the injury risk among patients with FAI increases with VD during drop landing, and 3) VD affects unstable extremities during drop landing in patients with FAI.

## Materials and methods

### Participants

A total of 12 males with FAI participated in the study, and all provided informed consent. The Soochow University Ethics Committee Board approved this study. The demographic information of the participants is shown in Table 1. Self-reported instability and function were determined using the ankle joint functional assessment tool (AJFAT) (Ross et al., 2008; Ibrahim and Abdallah, 2020). Participants’ inclusion criteria were as follows: 1) one unstable extremity, with AJFAT scores ≤26 (Delahunt et al., 2006; de Noronha et al., 2008; Kim et al., 2021; Rosen et al., 2021); 2) one stable extremity, with AJFAT scores >26; 3) no vestibular, visual, or neurological disease; 4) no lower extremity injuries for ≥6 months; and 5) only one side of the lower extremities suffering from FAI.

**TABLE 1:** Sociodemographic characteristics at baseline.

Characteristic		Patients with FAI (*n* = 12)
Age (years)		23.00 ± 0.78
Height (m)		1.68 ± 0.06
Weight (kg)		62.15 ± 10.44
Unstable extremity	Left	9/75%
	Right	3/25%

### Experimental procedure

**FIGURE 1:**
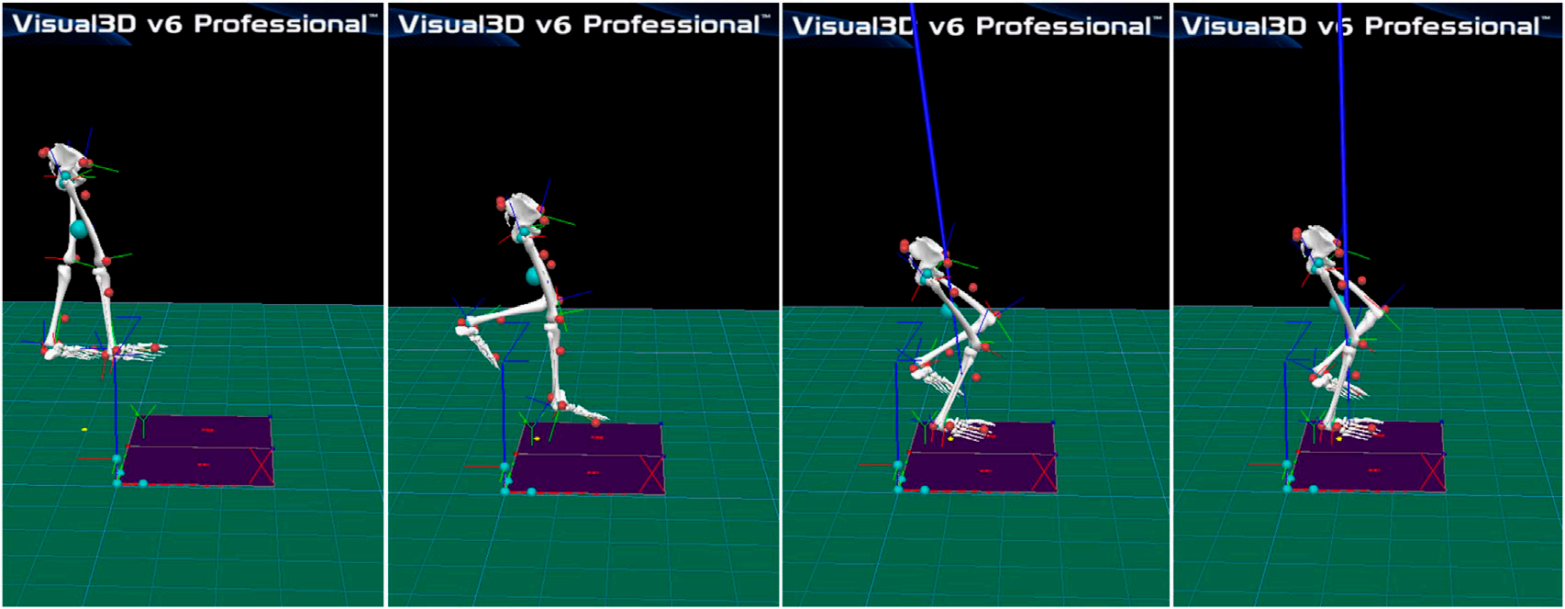
Participants landed toe first on one leg from a 30-cm-high step onto a force platform and were instructed to regain postural stability as quickly as possible.

Participants had had no strenuous activities or muscle fatigue within 24 h before the experiment. Initially, participants warmed up on a treadmill at 2.2 m/s for 3–5 min and stretched. In a single session, each participant with FAI wore experimental shorts and shoes and completed random drop landing trials without initial speed under four conditions, consisting of all combinations of each leg (unstable and stable extremities) and two conditions (VP and VD). Subsequently, participants completed a tiptoe slide from the platform’s edge at a height of 30 cm, with instructions to place the hands akimbo to reduce the influence of arm swing. Two examiners provided safety assistance throughout the experiment. Landing on a single leg and bending knees instinctively and toe-first is illustrated in Figure 1. The two visual conditions were tested randomly. The participants were required to wear black blindfolds when testing under VD conditions (Shoja et al., 2020).

Each condition repetition was recorded by one examiner, who was blinded to the AJFAT scores, with a rest of 30 s between landings until the participant had completed three successful trials. Successful trials were defined as those performed without any extraneous movement during the 3 s prior to the hop and with the participant subsequently remaining upright during the 2 s after the drop landing, with the tested extremity within the boundary.

### Data processing

Lower extremities’ kinetic data were collected at 100 Hz using a 3D motion capture system equipped with eight infrared cameras (Vicon Motion Analysis, United Kingdom) by tracking 18 infrared-reflective balls (reflective markers) of 14 mm diameter. Ground reaction force data were sampled at 1000 Hz using a 3D Kistler force measuring platform (model: 9287B, 90 cm × 60 cm×10 cm, Kistler, Inc., Switzerland). The plug-in gait was modeled using Visual3D (Version 6, C-Motion, Inc., United States) to calculate joint kinetics.

The following kinetic data were analyzed: ground reaction force (GRF, including vertical GRF [*vGRF*], medial GRF [*mGRF*], and lateral GRF [*lGRF*]), time to peak GRF (T_GRF, including time to peak vertical GRF [*T_vGRF*], time to peak medial GRF [*T_mGRF*], and time to peak lateral GRF [*T_lGRF*]), loading rate (LR), joint torque in the sagittal plane and the frontal plane, hip joint vertical length variation (ΔL), lower extremity stiffness (K_leg_), and ankle stiffness. LR was calculated using Eq. 1. K_leg_ was calculated using Eq. 2.

LR=VGRF/T_vGRF, (1)

$$K_{leg}=vGRF∕\Delta L.$$ (2)

### Statistical analysis

The normality of each variable was determined using the Shapiro–Wilk test. Descriptive and dependent measures were calculated as mean ± SD. Dependent measures were used to examine the interaction effects of groups (unstable and stable extremities of patients with FAI) and conditions (VP and VD) that served as the repeated measure. Simple effect analysis was performed when significant interactions were found. If the simple effect analysis suggested a statistically significant difference in a metric in both groups between VP and VD, then the difference between VP and VD in each group was determined using the independent samples *t*-test. All statistical analyses were performed using a statistical software package (IBM SPSS Statistics, IBM, NY, United States), and the level of statistical significance was set at 0.05.

## Results

### GRF, T_GRF, and LR

The experimental data are shown in Table 2. No significant interaction effects in the group were detected for GRF, T_GRF, and LR. Significant effects of the condition were detected for vGRF (*p* = 0.041), T_vGRF (*p* = 0.001), T_lGRF (*p* < 0.001), and LR (*p* = 0.001). A significant effect in the group was detected for T_lGRF (*p* = 0.004).

**TABLE 2:** GRF, T-GRF, and LR of the unstable and stable extremities during landing with and without visual deprivation.

Variable	Group		Group	Condition	Condition × group
	Unstable extremity	Stable extremity	*p*-value	*p*-value	*p*-value
*Direction of Ground Reaction Force (N)*					
Vertical GRF					
VP	3.45 ± 0.48	3.62 ± 0.36	0.221	0.041	0.184
VD	3.92 ± 0.52	3.91 ± 0.46			
Medial GRF					
VP	0.27 ± 0.04	0.29 ± 0.02	0.311	0.959	0.647
VD	0.27 ± 0.03	0.28 ± 0.02			
Lateral GRF					
VP	−0.30 ± 0.02	−0.29 ± 0.02	0.999	0.195	0.999
VD	−0.30 ± 0.02	−0.29 ± 0.03			
*Time to Peak Ground Reaction Force (ms)*					
T_vGRF					
VP	59.00 ± 4.11	59.33 ± 2.93	0.229	0.001	0.186
VD	54.25 ± 5.60	57.17 ± 2.44			
T_mGRF					
VP	60.75 ± 3.52	60.92 ± 1.73	0.909	0.085	0.588
VD	60.08 ± 3.68	59.67 ± 2.46			
T_lGRF					
VP	58.17 ± 3.71	60.42 ± 1.83	0.004	<0.001	0.180
VD	54.33 ± 3.20	58.33 ± 2.31			
*Loading rate* (*ms* ^*−1*^)					
VP	60.63 ± 8.56	61.12 ± 5.40	0.964	0.001	0.676
VD	66.69 ± 9.98	65.93 ± 7.18			

“−” represents the value of lGRF.

#### Joint torque

The experimental data are shown in Table 3. No significant interaction effect between the group and the condition was detected for joint torque. Significant effects of the condition were detected for hip extensor torque (*p* = 0.025), knee extensor torque (*p* = 0.016), hip varus torque (*p* = 0.025), and knee varus torque (*p* = 0.002). Significant effects in the group were detected for hip extensor torque (*p* = 0.022), ankle plantarflexion torque (*p* < 0.001), and ankle varus torque (*p* = 0.021).

**TABLE 3:** Joint torque (hip, knee, and ankle) in the sagittal and frontal planes during landing with and without visual deprivation.

Variable	Group		Group	Condition	Condition × group
	Unstable extremity	Stable extremity	*p*-value	*p*-value	*p*-value
*Joint torque in the sagittal plane (Nm/kg)*					
*Hip*					
VP	−1.40 ± 0.23	−1.16 ± 0.40	0.022	0.025	0.461
VD	−1.64 ± 0.41	−1.29 ± 0.33			
*Knee*					
VP	1.32 ± 0.20	1.15 ± 0.29	0.090	0.016	0.913
VD	1.49 ± 0.29	1.34 ± 0.30			
*Ankle*					
VP	−1.76 ± 0.16	−1.54 ± 0.27	<0.001	0.825	0.091
VD	−1.90 ± 0.26	−1.44 ± 0.29			
*Joint torque in the frontal plane (Nm/kg)*					
*Hip*					
VP	−0.66 ± 0.12	−0.69 ± 0.14	0.398	0.025	0.205
VD	−0.88 ± 0.23	−0.76 ± 0.25			
Knee					
VP	0.05 ± 0.34	0.21 ± 0.50	0.223	0.002	0.757
VD	0.37 ± 0.51	0.59 ± 0.35			
*Ankle*					
VP	1.53 ± 0.20	1.40 ± 0.24	0.021	0.291	0.153
VD	1.81 ± 0.34	1.36 ± 0.62			

“−” represents hip extensor torque, ankle plantarflexion torque, hip varus torque, and knee eversion torque.

#### Stiffness

The experimental data are shown in Table 4. A significant interaction was detected for ΔL, and a statistically significant difference in ΔL was found between VP and VD in both groups. The ΔL of the groups decreased with VD, and VD affected unstable and stable extremities (unstable extremity, 0.236 ± 0.003 to 0.118 ± 0.003, *p* < 0.001; stable extremity, 0.256 ± 0.003 to 0.127 ± 0.002, *p* < 0.001). The unstable extremity revealed a shorter ΔL than the stable extremity, whether under VD or VP conditions (VP, 0.236 ± 0.003 to 0.256 ± 0.003, *p* < 0.001; VD, 0.118 ± 0.003 to 0.127 ± 0.002, *p* < 0.001). Furthermore, significant effects of the condition were detected for K_leg_ (*p* < 0.001) and ankle stiffness (*p* = 0.002). Significant effects in the group were detected for K_leg_ (*p* = 0.035) and ankle stiffness (*p* < 0.001).

**TABLE 4:** Stiffness of FAI during landing with and without visual deprivation.

Variable	Group		Group	Condition	Condition × group
	Unstable extremity	Stable extremity	*p*-value	*p*-value	*p*-value
*ΔL (m)*					
VP	0.236 ± 0.003	0.256 ± 0.003	<0.001	<0.001	<0.001
VD	0.118 ± 0.003	0.127 ± 0.002			
*K*_*leg*_ (*BW/m*)					
VP	16.63 ± 2.19	14.56 ± 1.18	0.035	<0.001	0.693
VD	30.18 ± 3.20	28.52 ± 2.53			
Ankle stiffness (N*m/Δθ)					
VP	−0.038 ± 0.005	-0.032 ± 0.006	0.001	0.002	0.263
VD	−0.047 ± 0.011	-0.036 ± 0.004			

## Discussion

In this study, we focused on the effects of VD on patients with FAI during drop landing from the perspective of kinetics. We found differences between stable and unstable extremities in patients with FAI during drop landing. VD has a negative impact on patients with FAI, and the impact on the unstable extremity is more pronounced, which is consistent with our hypothesis.

### Differences in kinetics between unstable and stable extremities

The results show that the T_lGRF was earlier in the unstable extremity than in the stable extremity, suggesting that the protective strategy for the unstable extremity may need to be sufficiently activated. Our results are similar to those of previous studies of patients with FAI during single-extremity jump landings, in that the severity of ankle instability correlates with the earlier frontal stabilization time (Ross et al., 2008). Some scholars (Lephart et al., 1998; Svinin et al., 2019) have suggested mechanisms responsible for the earlier T_lGRF in the unstable extremities and that the severity of ankle instability correlates with the shorter time to frontal stabilization. The proprioceptors in the ankle provide joint position sensation and motion signals along the sensory nerves to the spinal cord, where other sensory neurons may process and then transmit them to the brain for further processing. The proprioceptors damaged by recurrent sprains in the unstable extremities of patients with FAI may cause delayed signal transmission resulting in abnormal feedback (Lephart et al., 1998; Eechaute et al., 2009; Song et al., 2016; Ludvig et al., 2017; Ibrahim and Abdallah, 2020). This abnormal feedback may not be able to activate the protective strategy for landing in a timely, effective manner to reduce the injury risk. Moreover, studies (Horak et al., 1990; Patla et al., 2002; Chaudhry et al., 2005; Delahunt et al., 2006; Ergen and Ulkar, 2008; Kabbaligere et al., 2017; Ibrahim and Abdallah, 2020) have suggested that movement impairment could be modified by correcting muscle activations. Impaired proprioception causes a delay in the conduction of the afferent signal, and the consequent delay in the efferent signal causes a delay in the corrective muscle activation, which leads to a higher injury risk in the unstable extremity.

Higher hip extensor and ankle torque in the sagittal and frontal planes were revealed in the unstable extremity as compared with the stable extremity. Our results are similar to research on the effect of proprioceptive training on postural stability, that is, they show that a higher hip extensor torque increases ankle sprain risk (Hietamo et al., 2021). Joint torque represents the buffering function of energy absorption on the ground reaction force through muscle strength. The higher the hip extensor torque is, the less GRF is absorbed through the muscles, which means that the role of muscle strength in reducing ground reaction force is minor; the higher the load is, the less the extremity is buffered during drop landing. Based on kinetic chain theories, insufficient hip muscle function increases the likelihood of uncontrolled ankle displacements and ankle injury.

Previous studies have showed poor postural stability in ankle sprain patients (Delahunt et al., 2006; Doherty et al., 2014). Postural stability decreases when the unstable extremity is forced to support itself during drop landing. Corresponding to our results, studies have found that K_leg_ is larger in the unstable extremity than in the stable extremity. A study related to ankle and knee proprioception found that postural swing and center of body mass changes increase among ankle-injured individuals compared with the uninjured group and that a larger K_leg_ response might increase postural swing and center of body mass changes during movement (Lephart et al., 1998). Some studies (Ito and Gomi, 2020) have suggested the mechanisms of postural swing and center of body mass changes, in which impaired proprioception in the unstable extremity makes accurate adjustment signal transmission for joint position sense abnormal, then blocks joint pressure and tension feedback and further affects the nerve fiber processing of sensory information, thus aggravating postural instability.

The greater ankle stiffness in the stable extremity compared with the unstable extremity also supports the view that the unstable extremity has a higher injury risk. Ankle stiffness reduces energy consumption caused by joint activity through the rigid lever, and stored energy is released to cushion the ground impact force; ankle stiffness can prevent joint instability during drop landing (Gao et al., 2022). Ludvig et al. (2017) has suggested mechanisms for deficient levels of ankle stiffness, in which proprioception is impaired in the unstable extremity, leading to the muscle not activating. The ligaments and tendons become slack due to recurrent ankle sprain, resulting in energy consumption caused by joint activity. The energy available for cushioning the ground impact force is reduced, causing higher injury risk.

### Effect of visual conditions on patients with FAI

The result of increased vGRF and LR under VD in our paper shows the higher impact force and injury risk in the lower extremities of patients with FAI during drop landing with VD (Rosen et al., 2013). Under VD, the protective strategy may not be sufficiently activated to buffer load and reduce injury risk. Our results show that earlier T_vGRF and T_lGRF appear under VP than under VD. Previous studies have found that visual conditions affect postural stability (Zipori et al., 2018), and our research further found that VD increases the risk of injury in patients with FAI. Under VD, the brain is unable to conduct environmental image analysis and deliver control signals generated by visual information transfer to achieve a series of postural control outputs, such as muscle tension adjustment and joint angle change.

Moreover, our results show deficits in postural control in patients with FAI, revealing that hip and knee torque in the sagittal and frontal planes increase with VD and that the hip and knee strategies mobilize when the ankle is unresponsive. This phenomenon shows that the dynamic defense system of the joint is further limited due to the proprioception impairment caused by the recurrent ankle sprain, which leads to abnormal ankle strategies in postural control (Alghadir et al., 2020). In our paper, neither the unstable nor the stable extremities buffered the load through ankle torque. This result could reflect the inability of the unstable extremity to overcome the loss of two sources of sensory information (Song et al., 2016): proprioception, which is altered due to repeated ankle sprain, and visual inputs, which are lost under VD. Palm et al. (2009) studied the effects of vision and hearing on postural stability and suggested that other senses, such as proprioception and vestibular sense, can compensate for postural instability when visual information input is abnormal. Zipori et al. (2018) also suggested that proprioception dependence in individuals increases when visual information inputs are abnormal. However, our paper found that even the stable extremity, without proprioception impairment, does not show special performance compared with the unstable extremity during VD. Therefore, as for the stable extremity, proprioception dependence is reduced under the long-term influence of the unstable extremity deficits (Eechaute et al., 2009; Song et al., 2016).

Our results show that K_leg_ is larger under VD than under VP, indicating an increased instability and injury risk in the lower extremities. As mentioned previously, a larger K_leg_ may increase center of body mass changes and postural swing during drop landing. Eechaute et al. (2009) have supported this view and suggested that the center of body mass is used to maintain balance during motion. Palm et al. (2009) suggested that when visual control information decreases, postural control ability decreases and the lower extremity injury risk increases accordingly. In addition, muscle stiffness regulation is the first reflex mechanism to adjust posture stability. Neurofeedback modulates systems and adjusts muscle tone to adapt to changes in the spatial environment. A possible explanation for the lower ankle stiffness under VD could be that missed visual inputs impede information transmission, which activates the muscles (McKeon and Hertel, 2008; Ibrahim and Abdallah, 2020). Miller et al. (2018) have further reported the importance of visual feedback, which may allow users to receive external joint position cueing, which is also conducive to sending the protection strategy’s activation signal. The motion system subsequently manipulates ankle positioning and stiffness. When patients with FAI lose their normal perception of the external environment, ankle stiffness cannot be adjusted correctly.

## Practical implications

Patients with FAI are highly dependent on vision in postural control. Vision plays an essential role in protective strategy activation for providing assistance information during drop landing among patients with FAI. The results reported here show that the ΔL adopted is larger under VP than under VD. The ΔL is the vertical length change of the hip, reflecting the cushioning ability of lower extremities to the ground impact force, which could be considered a hip strategy that patients with FAI enable. The visual system supplies the central nervous system with continuous information about the body’s position in the environment and activates the protection strategy. The hip strategy undertakes most of the buffering load tasks. Studies of postural strategies related to the loss of proprioception and vestibular function have found that the hip strategy is adopted more when joint instability occurs (Horak et al., 1990; Lee and Bo, 2021; Lee et al., 2022). Furthermore, the body mainly relies on the ankle strategy to adjust postural stability when interferences are few or when environmental information changes, but the hip strategy also dominates for large changes. Therefore, the human body prioritizes adjusting hip activities to reduce the load during drop landing, such as by increasing hip flexion to maintain postural stability. One study (Palm et al., 2009) has indicated, by analyzing biofeedback mechanisms through a trial of the effects of visual and auditory inputs on posture control, that postural stability increases with an increasing degree of visual control. The unstable extremity relies more on visual inputs for postural control than does the stable extremity. In support, studies related to visual use have suggested an explanation for postural control deficits in patients with FAI: their reliance on visual information has increased due to decreased somatosensory information from the ankle complex (Song et al., 2016; Nobusako et al., 2021). Our results are similar, and we found a shorter ΔL on the unstable and stable extremities under VD than under VP. Still, the unstable extremity was shorter, indicating that vision plays an important role in maintaining the postural stability of patients with FAI; the effect of VD on the unstable extremity is more significant, which means that the hip strategy on the unstable extremity is more difficult to activate through increasing hip and knee flexion. Furthermore, in our study, lower ankle stiffness, in which the ankle cannot form a more stable structure, was revealed under VD. However, a more vertical and protective landing strategy to minimize injury risk has been proposed that provides greater mechanical protection by generating a close-packed position in the ankle or by increasing hip flexion to provide stability for an uncertain landing. To sum up, increasing hip flexion to increase ΔL by deepening squat range might be an effective precaution in reducing injury risk in the unstable extremity during drop landing. Miller et al. (2018) have found that individuals with lower ankle stiffness would utilize a more protective landing strategy that increases hip and knee flexion to provide stability for an uncertain landing.

In addition, Ibrahim and Abdallah (2020) have suggested that proprioception training improves balance and shortens reaction time in postural stability, which further proves that the later T_lGRF in the stable extremity in our paper arises from self-protection. Hence, the extension of T_GRF can also be used as a breakthrough in precautions for patients with FAI. The current paper also mentions the benefits of repeated practice. As one repeats these activities, a feedforward mechanism occurs unconsciously through the activation of the stabilizing joint muscles and through the buffered load by activated muscle strategies absorbing energy.

## Limitations

As an indicator of postural stability, ankle muscle strength plays a vital role in postural stability. This limitation is also a characteristic of our paper, which evaluated only kinetics during this protocol, so the possibility of an unknown kinetic effect of the muscle activation response on the lower extremities cannot be determined. Therefore, surface electromyography (sEMG) could be combined in future research on patients with FAI under VD to validate the dependence on vision and to determine what strategies the muscles take, whether protection strategies through the muscles are adopted by the unstable extremity to reduce the risk of injury, and whether warming up in advance can help muscles to activate faster as a precaution against ankle injury.

## Conclusion

Unstable extremities have additional injury risks compared with stable extremities in patients with FAI, and vision plays an important role in maintaining postural stability in these patients. VD increases the injury risk for both the unstable and stable extremities, but the effect on the unstable extremities is more significant and more likely to result in injury, as the control of the central nervous system on hip strategy is also affected. Deepening the squat range and avoiding prolonged reaction time might be effective precautions for reducing injury risk in patients with FAI during drop landing. In the future, muscle strength and training could be studied and combined with sEMG to evaluate precautions from a muscular perspective.

## Data Availability

The original contributions presented in the study are included in the article/Supplementary Material; further inquiries can be directed to the corresponding authors.
